# Characteristics and metabolic patterns of soil methanogenic archaea communities in the high‐latitude natural forested wetlands of China

**DOI:** 10.1002/ece3.7842

**Published:** 2021-07-04

**Authors:** Di Wu, Caihong Zhao, Hui Bai, Fujuan Feng, Xin Sui, Guangyu Sun

**Affiliations:** ^1^ Key Laboratory of Saline‐Alkali Vegetation Ecology Restoration (Northeast Forestry University) Ministry of Education Harbin China; ^2^ College of Life Science Northeast Forestry University Harbin China; ^3^ Key Laboratory of Fast‐Growing Tree Cultivating of Heilongjiang Province Forestry Science Research Institute of Heilongjiang Province Harbin China; ^4^ Heilongjiang Provincial Key Laboratory of Ecological Restoration and Resource Utilization for Cold Region School of Life Sciences Heilongjiang University Harbin China

**Keywords:** community diversity, indicator species, methanogenic metabolic patterns, methanogens, wetlands

## Abstract

Soil methanogenic microorganisms are one of the primary methane‐producing microbes in wetlands. However, we still poorly understand the community characteristic and metabolic patterns of these microorganisms according to vegetation type and seasonal changes. Therefore, to better elucidate the effects of the vegetation type and seasonal factors on the methanogenic community structure and metabolic patterns, we detected the characteristics of the soil methanogenic *mcr*A gene from three types of natural wetlands in different seasons in the Xiaoxing'an Mountain region, China. The results indicated that the distribution of Methanobacteriaceae (hydrogenotrophic methanogens) was higher in winter, while Methanosarcinaceae and Methanosaetaceae accounted for a higher proportion in summer. Hydrogenotrophic methanogenesis was the dominant trophic pattern in each wetland. The results of principal coordinate analysis and cluster analysis showed that the vegetation type considerably influenced the methanogenic community composition. The methanogenic community structure in the *Betula platyphylla*–*Larix gmelinii* wetland was relatively different from the structure of the other two wetland types. Indicator species analysis further demonstrated that the corresponding species of indicator operational taxonomic units from the *Alnus sibirica* wetland and the *Betula ovalifolia* wetland were similar. Network analysis showed that cooperative and competitive relationships exist both within and between the same or different trophic methanogens. The core methanogens with higher abundance in each wetland were conducive to the adaptation to environmental disturbances. This information is crucial for the assessment of metabolic patterns of soil methanogenic archaea and future fluxes in the wetlands of the Xiaoxing'an Mountain region given their vulnerability.

## Graphical abstract

To better elucidate the effects of the vegetation type and seasonal factors on the methanogenic community structure and metabolic patterns, we detected the characteristics of the soil methanogenic *mcr*A gene from three types of natural wetlands in different seasons in the Xiaoxing'an Mountain region, China. The results indicated that the distribution of Methanobacteriaceae (hydrogenotrophic methanogens) was higher in winter, while Methanosarcinaceae and Methanosaetaceae accounted for a higher proportion in summer. Hydrogenotrophic methanogenesis was the dominant trophic pattern in each wetland. Network analysis showed that cooperative and competitive relationships exist both within and between the same or different trophic methanogens. This information is crucial for the assessment of metabolic patterns of soil methanogenic archaea and future fluxes in the wetlands of the Xiaoxing'an Mountain region given their vulnerability.

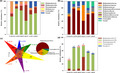

## 1 INTRODUCTION

Wetlands, as one of the most important ecosystems, play an important role in the global carbon cycle (Sjogersten et al., 2014) and are a major source of methane (CH_4_) in the atmosphere, accounting for about 30%–50% of the global emissions (Bridgham et al., 2013; George et al., 2020). The emission of methane from wetlands, which involves a variety of physical, chemical, and biological reactions, is a complex process, and it is primarily controlled by soil temperature, redox potential, availability of carbon substrates, soil texture, salinity, and land use types as well as soil microorganisms (Lyu et al., 2018; Martin et al., 2018; Poffenbarger et al., 2011; Taumer et al., 2021; Zhang, Luo, et al., 2018). The disturbance to forested wetland (human‐induced or natural) can lead to alterations in the composition of plant community and wetland ecosystem function, and potentially influence greenhouse gas emissions (Beach et al., 2009; Jancoski et al., 2019; Reddy et al., 2015). At present, it is practicable to control methane emissions in wetlands by adjusting water table depth, changing plant community composition, decreasing water eutrophication, and regulating the interaction of soil pH with mean annual air temperature (Abdalla et al., 2016; Martin et al., 2018). Furthermore, a particular concern is the role of methanogens as a biological factor in the release of methane from these ecosystems to the atmosphere.

Methanogens play an essential role in regulating global carbon cycle, especially CH_4_ emission (Zhang et al., 2020). Methanogenesis is an anaerobic decomposition process that forms the terminal step and is implemented by members of the phylum Euryarchaeota (Liu & Whitman, 2008; Zhang, Luo, et al., 2018). In wetlands, there are two primary groups of methanogens: acetoclastic methanogens and hydrogenotrophic methanogens (Conrad, 2007). There are differences in the metabolic pathways of the phylogenetic groups of methanogens. Some of them only use one pathway, while the others can utilize two (Godin et al., 2012). The complex interactions of soil methanogenic communities influence wetland ecosystem structures and functions (Deng et al., 2012; Li et al., 2021). A previous study found that the coexistence patterns of soil methanogenic communities in rice paddies are closely associated with their function, and their complex interactions may contribute more to soil functions, compared with species diversity (Li et al., 2021). Thus, an understanding of methanogenic interspecies interactions in complex microbial ecosystems is essential for recognizing both sharing and competition among methanogens in similar trophic niches (Kato et al., 2014).

Plant composition has been identified as a main factor regulating rates of CH_4_ emissions (Gutenberg et al., 2019; Ward et al., 2013). Shifts in plant communities can have a large influence on the activity and community composition of methanogens via inducing changes in environmental conditions or in the substrates available for root exudation from photosynthate (Merila et al., 2006; Wu et al., 2012). Plant roots, by producing secondary macropores, can strengthen soil aeration, change the redox potential in root environments, and ultimately influence methane emissions (Ndanga et al., 2016). In addition, temperature is considered a key factor affecting seasonal and annual variations in CH_4_ fluxes in wetlands (Bloom et al., 2012; Chen et al., 2013; Zhang, Luo, et al., 2018). Soil methanogen communities have made a feedback on climate change (Kim et al., 2012). Previous studies have demonstrated that temperature could influence the structure of methanogens from wetland soil (Fu et al., 2015), peatlands (Kim et al., 2012; Martí et al., 2015), and subarctic permafrost (Metje & Frenzel, 2007). Furthermore, the diversity of methanogen species is a potential predictor of CH_4_ production (Godin et al., 2012).

Forested wetlands are an important component of wetland environments. They are located at the interface between forest and water ecosystems, and have more complex service functions than either wetlands or forests (Li & Zeng, 2017; Yang et al., 2017). The wetlands of Xiaoxing'an Mountain, China, whose total area is 1,069,600 m^2^, are representative of the main mountainous wetlands found in high‐latitude temperate areas and play a critical role in the study of Chinese wetlands (Sun et al., 2009). Wetlands in this area are diverse and include forested wetland, shrub wetland, herbaceous vegetation wetland, moss bog wetland, and shallow marshland, most of which are primarily forested wetland types. Currently, there is a lack of understanding regarding the indicative groups and potential metabolic patterns of the methanogenic community in the high‐latitude wetlands of China's forested areas.

In this study, three typical natural wetlands along the forest–wetland transition zone of Xiaoxing'an Mountain, namely the *Betula platyphylla*–*Larix gmelinii*, *Alnus sibirica*, and *Betula ovalifolia* wetlands, were selected. The protein‐encoding gene *mcr*A was chosen as a molecular marker using high‐throughput sequencing to (a) explore the distribution characteristics of soil core methanogenic communities in the three wetlands; (b) elucidate the divergence of the methanogenic community among different vegetation types and predict the potentially methanogenic metabolic patterns in wetlands; and (c) determine the indicator species of soil methanogens according to vegetation type and seasonal variability. Exploring the community distribution characteristics of methanogens in the transitional zone of forested wetland is important for the prediction of methane release during natural forest‐wetland vegetation succession in northeastern of China.

## 2 MATERIALS AND METHODS

### 2.1 Study site and sampling

The study area is located in Wuyiling Wetland Nature Reserve of Xiaoxing'an Mountain in Northeastern China (E129°00′–129°30′, N48°33′–48°50′). The terrain of this area is characterized by modestly undulating, low hills with an altitude of 350–550 m. The zonal vegetation type is conifer–broadleaf forest dominated by *Pinus koraiensis*. Its climate belongs to the temperate continental monsoon climate, with an average annual precipitation of 584 mm. The annual average temperature is −1.1°C, and annual accumulated temperature (≥10°C) is 1,700–2,000°C. The frost‐free period is approximately 97 days, and the freezing period is 180 days. The snow accumulation period is approximately 160 days, and the average snow depth is 27 cm. The zonal soil type is dark brown soil, while azonal soils include meadow soil, swamp soil, and peat soil (Cai et al., 2010).

One study site was set up in each of the following wetland types: *B*. *platyphylla*–*L. gmelinii* (a forested wetland, abbreviated as BLW), *A. sibirica* (a forested wetland; ASW), and *B. ovalifolia* (a shrub wetland; BOW). Three 20 × 20 m sampling plots were established at least 50‐m intervals within each study site. Soils from each plot were sampled in winter (December 2015) and summer (August 2016). After the litter layer was removed, samples from each plot were collected with the upper layer of soil (0–10 cm) using a 10‐spot sampling method and mixed. After sieving, the samples were stored at −80°C until DNA extraction for soil methanogenic community analysis was commenced.

### 2.2 DNA extraction and *mcr*A gene amplification

Total DNA was extracted from the soil samples using a Power Soil DNA Isolation Kit (MOBIO Laboratories Inc.) according to the manufacturer’s instruction. MLF (5′‐GGTGGTGTMGGATTCACACARTAYGCWACAGC‐3′) and MLR (5′‐TTCATTGCRTAGTTWGGRTAGTT‐3′) primers were used to amplify the methanogenic *mcr*A gene (Luton et al., 2002). After initial denaturation at 95°C for 3 min, a PCR reaction was carried out with 39 cycles at 95°C for 30 s, 51°C for 30 s, 72°C for 45 s, with a final extension at 72°C for 10 min. Each primer pair was tagged using a unique barcode. Amplicon sequencing was performed on an Illumina MiSeq PE 300 platform according to standard protocols.

### 2.3 Illumina sequencing and data analysis

The raw sequencing data of the methanogenic *mcr*A gene amplicons were optimized, filtered, and assessed using Trimmomatic (Bolger et al., 2014). Forward and reverse reads were merged using Flash (version 1.2.11). Sequences with an average quality score <20 were filtered, while sequences that contained ambiguous (N) bases were screened out (Xu et al., 2017). Exact barcode matching was employed, with a two‐nucleotide mismatch permitted for primer matching. The trimmed sequences with chimeras were removed using the Uchime algorithm (Sui et al., 2019). To equalize the read sizes of the samples, each sample was downsized to 48,929 reads by random selection for further standardization analysis. The sequences were clustered into operational taxonomic units (OTUs) by setting a sequence similarity standard of 97%. A representative sequence for each OTU was assigned to analyze taxonomic information based on the functional gene database (http://fungene.cme.msu.edu/). The α‐diversity indices including Shannon index, Simpson, Chao estimator, ACE, and the Good’s coverage estimator were calculated using Mothur at 97% identity.

### 2.4 Statistical analysis

One‐way analysis of variance (ANOVA) was used to test the significance among the treatments on these alpha‐diversity indices in SPSS 16.0 (SPSS Inc.). Tukey’s multiple comparison test was then applied to determine the significance of the differences at a *p*‐value of .05. A significance test of relative abundance at class and family level was conducted using STAMP Software (Parks et al., 2014). A Venn diagram was used to show the common and unique OTUs using R software (Venn diagram package). A principal coordinate analysis (PCoA) of OTUs of all samples was generated based on the Bray–Curtis dissimilarity distance test. The soil methanogenic feature of the three vegetation types was determined in order to identify the significant biomarkers using linear discriminate analysis effect size (LEfSe) analysis (LDA scores = 2) (Ling et al., 2016).

A co‐occurrence network analysis was built using the relative abundance of top 60 OTUs with Spearman’s rank correlation (coefficient *r* ≥ .7 and *p* < .05). The network was visualized using Cytoscape (version 3.7.1) (Shannon et al., 2003). The hierarchical cluster of all samples was constructed alongside the Bray–Curtis dissimilarity distance matrix at the OTU level using the R software’s Vegan package. The permutational multivariate analysis of variance (PERMANOVA; 999 permutations) further assessed the significant differences within the hierarchical cluster of methanogenic communities.

Indicator species analysis was conducted using the “labdsv” and “vegan” packages in R software. For this analysis, all OTUs were selected. Species with a high indicator value (IndVal; established using a Monte Carlo permutation with 1,000 replicates) were considered the most pivotal indicators within the community (De Cáceres et al., 2010). Finally, a phylogenetic tree was constructed using the relative abundance of the top 500 species. This was calculated based on the maximum likelihood method and computed using FastTree software (version 2.1.3).

## 3 RESULTS

### 3.1 Richness and diversity indices

The α‐diversity indices were analyzed based upon the *mcr*A gene data for the different vegetation types and seasons (Table 1). The Good’s coverage estimator ranged from 99.95% to 99.99% for all samples, indicating that the sequences obtained represent the dominant phylotypes. In total, 147–496 OTUs were detected in different treatments, which implies that methanogen species are distributed widely in these wetland soils. Overall, the numbers of methanogen OTUs in winter were generally slightly higher than those in summer, compared with corresponding vegetation type. Except for *B. platyphylla*–*L. gmelinii* wetland in winter, the number of methanogenic OTUs in the *A. sibirica* wetland was slightly above that found in the other wetlands, in either winter or summer.

**TABLE 1:** Alpha‐diversity indices of soil methanogen communities from the three surveyed wetland sites

Samples	OTUs		Shannon		Simpson		ACE		Chao		Good's coverage	
	Mean	*SE*	Mean	*SE*	Mean	*SE*	Mean	*SE*	Mean	*SE*	Mean	*SE*
W‐BLW	496	366	3.69	0.64	0.08a	0.01	505.23	365.36	509.55	368.34	99.95%	0.03%
W‐ASW	274	88	3.58	0.15	0.06a	0.01	289.12	83.54	295.29	79.86	99.95%	0.01%
W‐BOW	210	117	3.49	0.11	0.06a	0.01	223.13	113.66	224.19	114.68	99.96%	0.02%
S‐BLW	147	88	2.97	0.27	0.13b	0.03	149.61	89.84	149.81	91.56	99.99%	0.01%
S‐ASW	211	66	3.68	0.04	0.05a	0.002	220.46	72.74	224.94	77.63	99.97%	0.02%
S‐BOW	158	29	3.25	0.11	0.06a	0.01	170.56	26.11	177.49	18.67	99.96%	0.01%
*F*	1.788		2.704		9.362		1.808		1.798		1.678	
*P*	.190		.073		.001		.186		.188		.214	

Note

The different letters indicate a significant difference among the different wetlands under different seasons as determined by Tukey’s multiple comparison test.

Abbreviations: ASW, *A*. *sibirica* wetland; BLW, *B. platyphylla*–*L. gmelinii* wetland; BOW, *B*. *ovalifolia* wetland; S, summer; SE, standard error; W, winter.

Similar trends were observed in the results of the Shannon, ACE, and Chao indices, which also showed that, in each vegetation type, the diversity and richness of methanogens clearly change with season. In addition, the Simpson's evenness index revealed that the distribution of the methanogen community in the *B. platyphylla*–*L. gmelinii* wetland was very uneven compared with that of the other wetlands. A one‐way ANOVA of each index demonstrated that the results of the diversity and richness indices (including the Shannon, ACE, and Chao indices) were not significantly different (Table 1), but the results of the Simpson index were significantly different across vegetation type (*p* = .001).

### 3.2 Taxonomic characteristics of methanogenic communities

**FIGURE 1:**
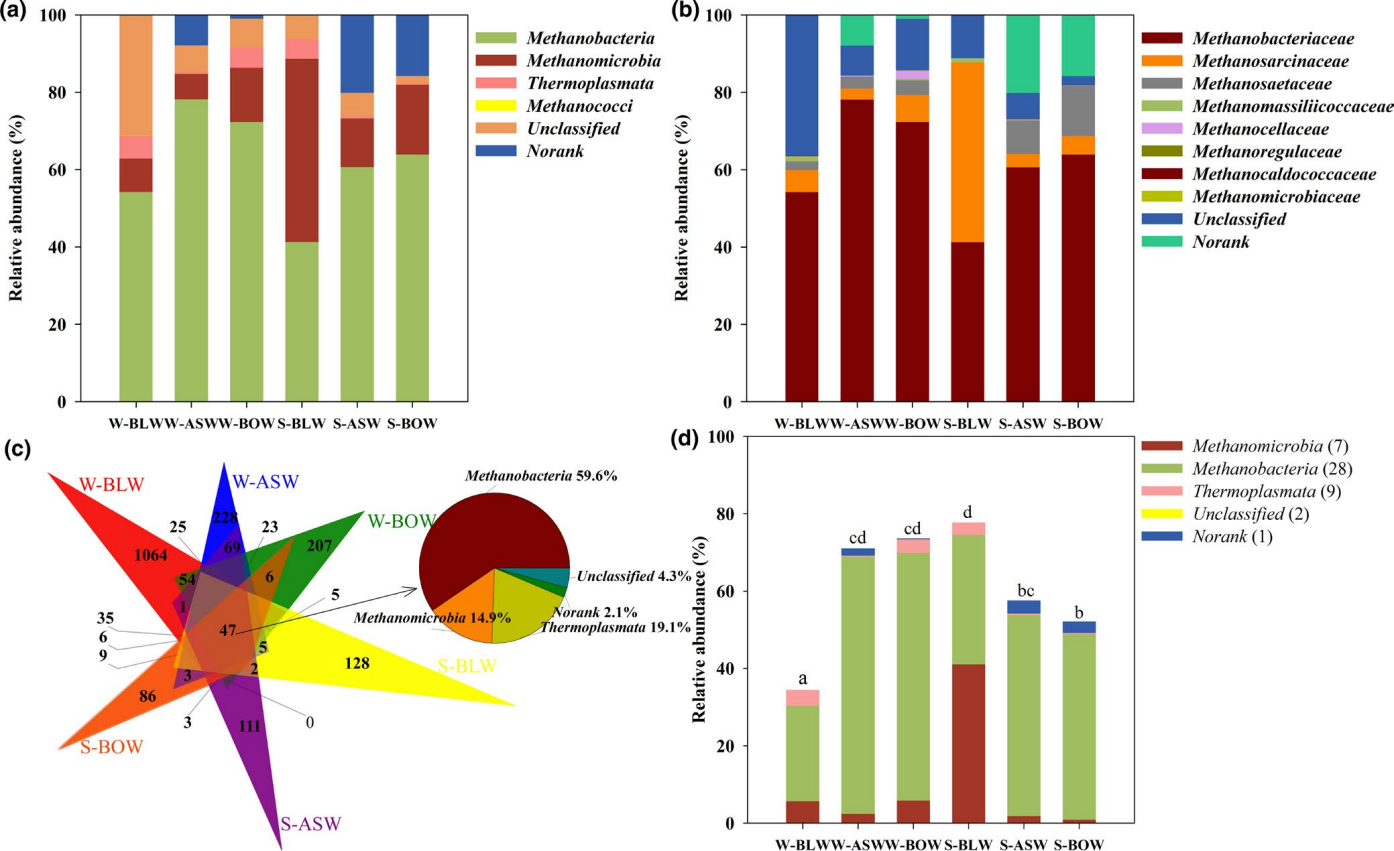
Bar charts of the relative abundance at methanogenic (a) class level and (b) family level. (c) Venn diagram displaying the number of unique and common OTUs in all samples. (d) Bar chart of the relative abundance of the core OTUs (consisting of 47 common OTUs) at class level in different wetland soils. The different letters indicate a significant difference in the total relative abundance of core OTUs among the different wetland types as determined by Tukey’s multiple comparison test

Using taxonomic analysis at the class level, all OTUs obtained were identified as belonging to four taxonomic classes known to be present in all wetlands (Figure 1a). The dominant classes in each of the three wetland types were Methanobacteria, Methanomicrobia, and Thermoplasmata, all of which belong to Euryarchaeota. Many differences in the community characteristics of these dominant classes were present in each wetland. The proportion of Methanobacteria was higher in winter in all wetlands, whereas Methanomicrobia had a higher distribution in summer compared with the corresponding wetland. Furthermore, the proportion of the most dominant class Methanobacteria was lower in the *B. platyphylla*–*L. gmelinii* wetland than in the other wetlands. In this habitat, its proportion dropped to 41.3% in summer, during which the proportion of Methanomicrobia remarkably increased (Figure S1). Moreover, the highest number of unclassified OTUs was detected in the *B. platyphylla*–*L. gmelinii* wetland in winter, while the proportion was lowest in the *B. ovalifolia* wetland in summer.

At the family level, the effective sequences could be mostly classified into eight taxonomic families (Figure 1b). Methanobacteriaceae, Methanosarcinaceae, and Methanosaetaceae were the most abundant families in all soil samples. Methanobacteriaceae accounted for a higher proportion (54.2%–78.1%) in winter, while the proportions of Methanosarcinaceae and Methanosaetaceae increased gradually in summer. Noticeably, the total number of effective sequences assigned to Methanosarcinaceae in the *B. platyphylla*–*L. gmelinii* wetland in summer was significantly higher than that of the other wetlands (Figure S2). Additionally, several small parts of the sequences could not be identified at the family level, which implies that unknown methanogens may exist in the wetlands studied.

Despite the clear differences at class and family levels, heterogeneity needs to be analyzed further at lower taxonomic levels. A Venn diagram was drawn to illustrate the common and unique methanogenic OTUs present in all three wetland types studied (Figure 1c). The methanogenic communities of all soil samples shared only 47 common OTUs, most of which can be assigned to Methanobacteria (59.6%), Thermoplasmata (19.1%), and Methanomicrobia (14.9%). These 47 OTUs were considerably abundant, together taking up 34.5%–77.7% of the relative abundance at the class level in the whole community of each wetland, while the relative abundance of these core OTUs in *B. platyphylla*–*L. gmelinii* wetland in summer was the highest (Figure 1d and Table S1). Furthermore, the Venn diagram showed that the *B. platyphylla*–*L. gmelinii* wetland had the highest number of unique OTUs in winter, while the *B. ovalifolia* wetland had only 86 unique OTUs in the summer. Overall, every wetland observed a higher number of unique methanogenic OTUs in winter than in summer.

**FIGURE 2:**
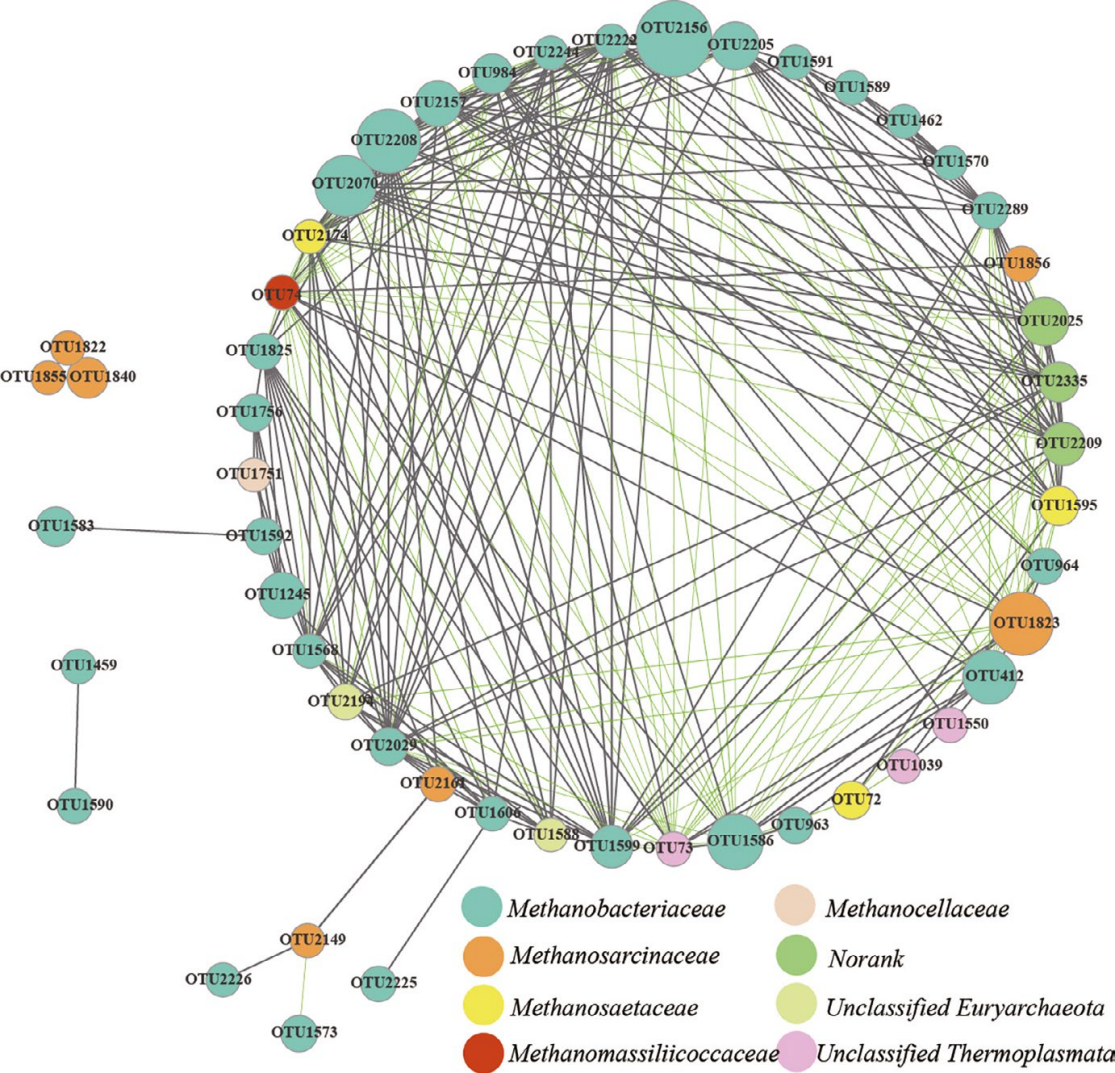
Network analysis revealing the connection pattern of the soil methanogenic community according to wetland type and season. The top 60 OTUs were selected according to mean abundance from all samples. The size of each node is proportional to the relative abundance of methanogenic OTUs. Nodes from the same family in the network are marked with identical colors. The thickness of each edge with *r* ≥ .7 and *p* < .05 is proportional to the coefficient value (*r*)

Network analysis was performed to assess the relationships among methanogenic communities at OTU level (Figure 2). The dominant methanogenic OTUs (51 nodes) were chosen to identify the positive and negative relationships (276 edges) in the network. Out of a total of 276 edges, 185 represented positive connections, while 91 expressed negative connections. These dominant OTUs belonged to eight families, with Methanobacteriaceae being the most abundant. Thus, the existence of interrelated edges suggests that an important connection between Methanobacteriaceae and other families exists in the co‐occurrence network.

### 3.3 Comparison of methanogenic community similarities

To analyze the similarity of the methanogenic communities, cluster analysis was applied based upon the Bray–Curtis dissimilarity distance test (Figure S3). In the results, soil samples from the *B. ovalifolia* and *A. sibirica* wetlands were gathered in a cluster, which implies that the methanogenic communities of these wetland types are similar. Soil samples from the *B. platyphylla*–*L. gmelinii* wetland formed an independent branch, indicating that the methanogen community in this wetland is distinctly differentiated from the other cluster. According to PERMANOVA analysis, there was a significant difference between the two clusters (*r* ^2^ = .384, *p* = .001).

**FIGURE 3:**
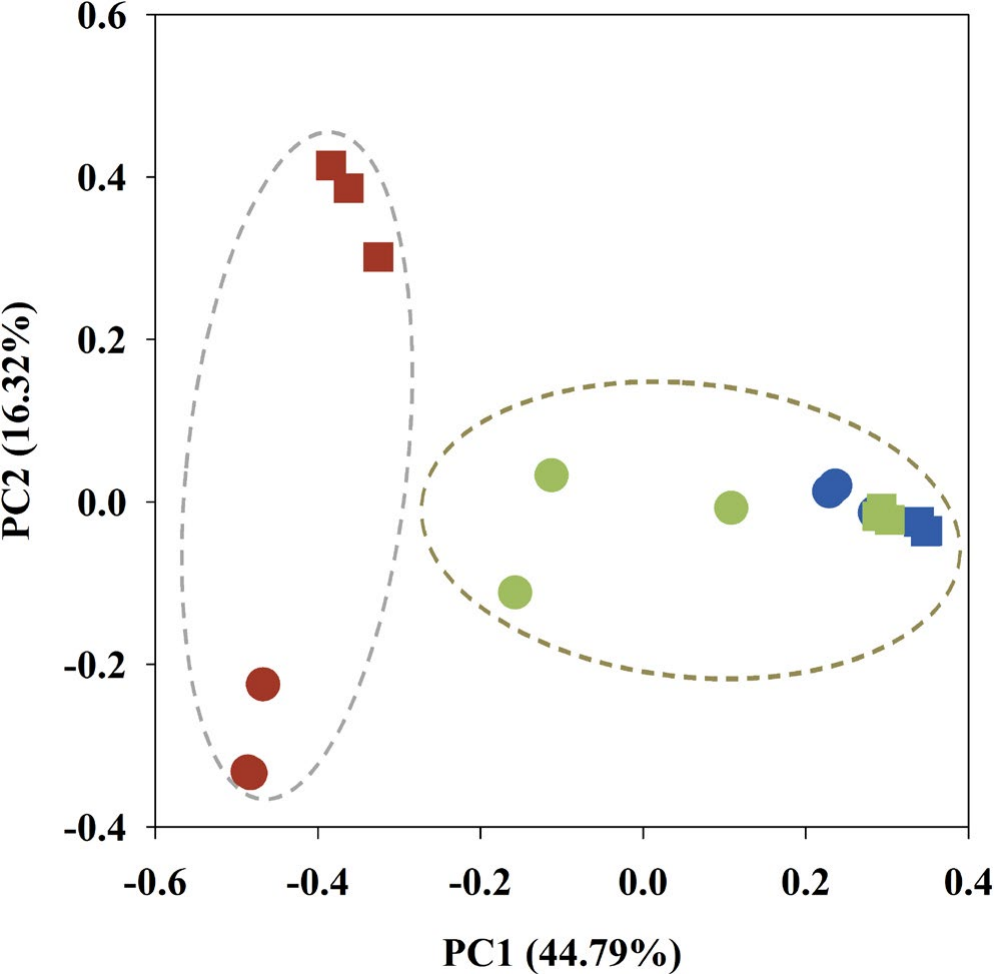
Principal coordinate analysis of the methanogenic communities found in three different wetland types. Circles represent winter, while squares represent summer. Red represents *Betula platyphylla*–*Larix gmelinii* wetland, blue represents *Alnus sibirica* wetland, green represents *Betula ovalifolia* wetland

To further verify the results from cluster analysis, principal component analysis was performed using uniFrac PCoA based on the OTUs of the *mcr*A gene (Figure 3). The first two PC axes were estimated on account of the results from the soil samples, with 44.79% and 16.32% for the variance, respectively. Two groups were detached from each other along the PC1 axis in the PCoA diagram. The *B. platyphylla*–*L. gmelinii* wetland samples from both seasons were clustered together, while the samples of the *B. ovalifolia* and *A. sibirica* wetlands were combined into a group and spread along the positive direction of the PC1 axis. The pattern of the PCoA further strengthened the result of the cluster analysis. At the same time, it can be seen that the distribution of the methanogen communities was primarily characterized according to vegetation type.

**FIGURE 4:**
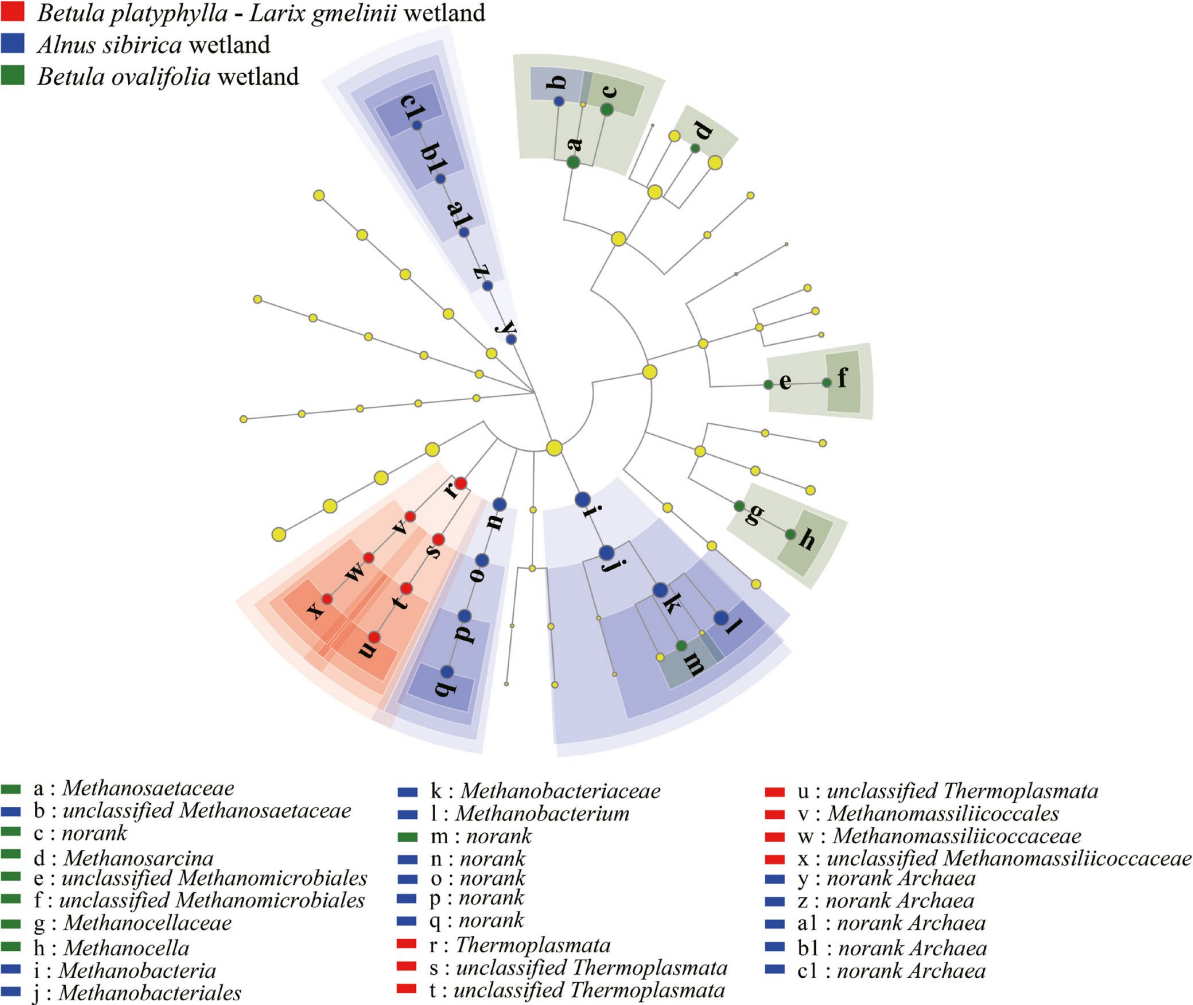
Taxonomic cladogram of LEfSe analysis from phylum to genus level of the three different vegetation types

Considering the aforementioned results, LEfSe analysis was used to distinguish the potential discriminating taxa from the methanogenic communities in each wetland type. The larger differences in the taxa were depicted using a cladogram (Figure 4). The LEfSe analysis showed that there were 29 significantly differential taxa across the three vegetation types (LDA score = 2). Most taxa were attributed to the *A. sibirica* wetland (14 taxa), followed by the *B. ovalifolia* wetland (eight taxa), and the *B. platyphylla*–*L. gmelinii* wetland (seven taxa). In this cladogram, most of the taxa in the *A. sibirica* wetland were no rank or unclassified except for the members of Methanobacteria. The taxa in the *B. platyphylla*–*L. gmelinii* wetland can be primarily assigned to class Thermoplasmata, while those in the *B. ovalifolia* wetland belonged to the Methanocellaceae and Methanosaetaceae families.

### 3.4 Responses of methanogenic indicator species to vegetation type and season

To determine the influence of vegetation type and season on methanogens, indicator species analysis was performed for all OTUs. This analysis demonstrated that 28 OTUs were significantly associated with vegetation type and seasonal pattern, as listed in Table 2. Five OTUs represented the *A. sibirica* wetland in winter and were assigned to the Methanosaetaceae and Methanobacteriaceae families, while two indicator OTUs were representative of the summer. Only two indicator OTUs were found in the winter *B. platyphylla*–*L. gmelinii* wetland data, whereas there were nine for the summer. The indicator OTUs that were associated with the *B. ovalifolia* wetland for the summer all belonged to the family Methanosaetaceae (three OTUs), while the seven associated with the winter were classified into Methanosaetaceae and Methanobacteriaceae.

**TABLE 2:** Methanogenic indicator OTUs for different vegetation types and seasons (IndVal > 0.6, *p* < .05)

Group	OTUs	IndVal	*p*‐Value	Taxa/Classification	Group	OTUs	IndVal	*p*‐Value	Taxa
Winter					Summer				
BLW	OTU14	1	.001	Unclassified Methanomicrobia	BLW	OTU1854	1	.003	Unclassified Thermoplasmata
BLW	OTU1586	0.70	.016	Methanobacteriaceae	BLW	OTU1822	0.99	.004	Methanosarcinaceae
ASW	OTU1567	0.90	.002	Methanosaetaceae	BLW	OTU1855	0.99	.001	Methanosarcinaceae
ASW	OTU2244	0.79	.001	Methanobacteriaceae	BLW	OTU1846	0.98	.001	Unclassified Methanocellales
ASW	OTU1524	0.77	.001	Methanobacteriaceae	BLW	OTU1847	0.90	.001	Unclassified Thermoplasmata
ASW	OTU1597	0.72	.001	Methanobacteriaceae	BLW	OTU1845	0.88	.007	Methanosarcinaceae
ASW	OTU1606	0.67	.009	Methanobacteriaceae	BLW	OTU1823	0.82	.014	Methanosarcinaceae
BOW	OTU1542	0.95	.026	Unclassified Methanomicrobiales	BLW	OTU2226	0.75	.001	Methanobacteriaceae
BOW	OTU1544	0.89	.006	Methanobacteriaceae	BLW	OTU1234	0.74	.022	Unclassified Methanocellales
BOW	OTU2229	0.68	.009	Methanobacteriaceae	ASW	OTU2255	0.83	.016	Methanosaetaceae
BOW	OTU1462	0.65	.01	Methanobacteriaceae	ASW	OTU2174	0.80	.011	Methanosaetaceae
BOW	OTU1591	0.75	.014	Methanobacteriaceae	BOW	OTU2294	0.97	.002	Methanosaetaceae
BOW	OTU1579	0.89	.02	Methanosaetaceae	BOW	OTU1595	0.79	.004	Methanosaetaceae
BOW	OTU1576	0.82	.008	Methanosaetaceae	BOW	OTU2126	0.68	.005	Methanosaetaceae

**FIGURE 5:**
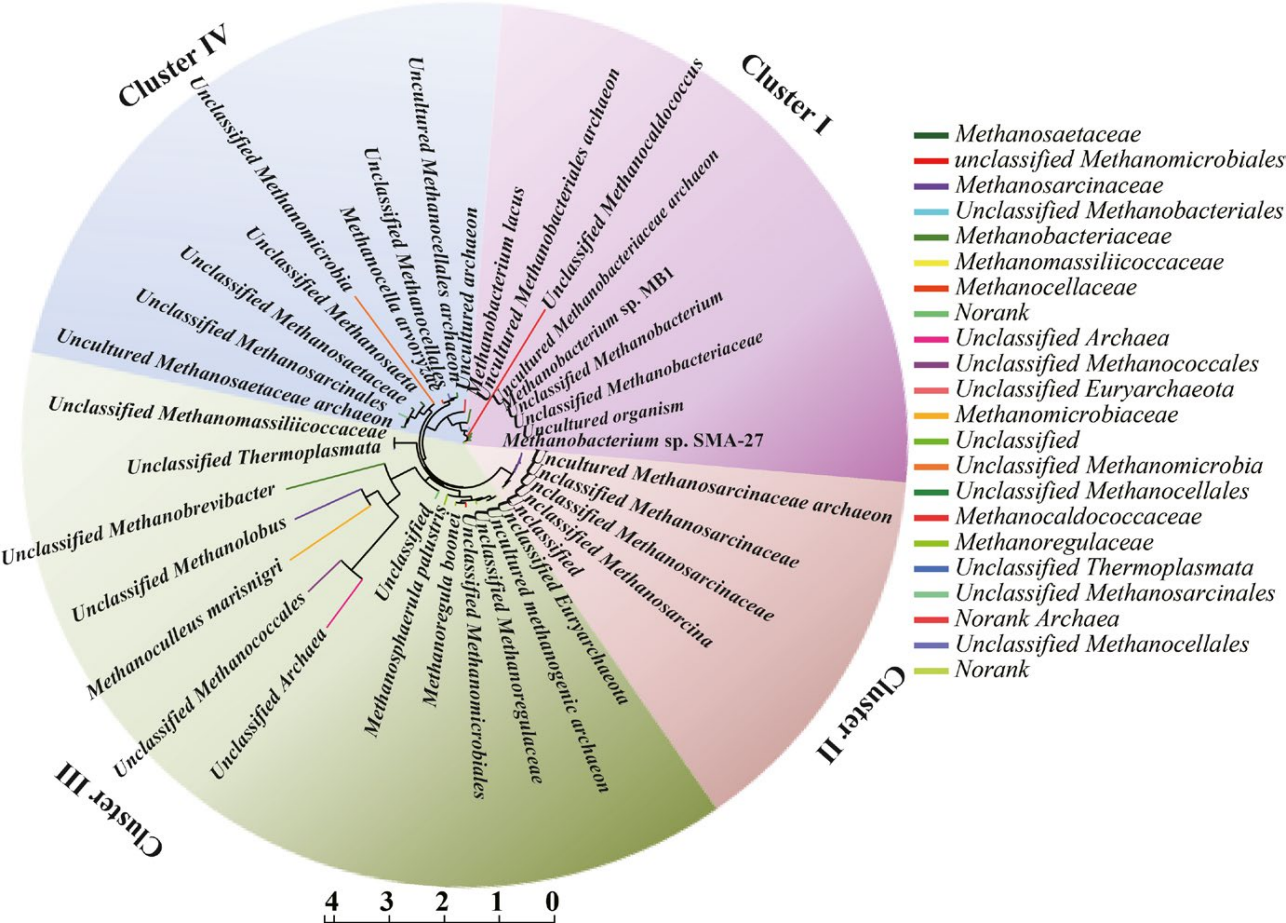
Phylogenetic affiliations of methanogenic species from different wetland soils

Coevolution of the methanogens is clearly demonstrated by the similar tree topologies of the *mcr*A gene (Figure 5). Based on phylogenetic analysis, the methanogenic species of different wetland soils were categorized into four clusters (I, II, III, and IV). Cluster I contains the representative species assigned to *Methanobacterium lacus*, *Methanobacterium* sp. MB1, and *Methanobacterium* sp. SMA‐27. Cluster II represents an unclassified cluster of methanogens within Methanosarcinaceae. Cluster III includes *Methanoregula boonei*, *Methanosphaerula palustris*, and *Methanoculleus marisnigri*, and the unclassified Thermoplasmata cluster. Cluster IV included not only the divergent hydrogenotrophic Methanocellales but also the unclassified Methanosaetaceae and Methanosarcinales.

## 4 DISCUSSION

In this study, we demonstrated that the structure and diversity of methanogenic communities in different wetland soils vary with season. Shifts in vegetation type and season correspond to variation in the indicator taxa and metabolic patterns of methanogenic communities. Here, we focused on the roles that vegetation type and seasonal mixing played in structuring methanogenic communities.

Forty‐seven common OTUs, mostly assigned to the known classes of Methanobacteria (59.6%), Thermoplasmata (19.1%), and Methanomicrobia (14.9%), were detected in all soil samples in the Venn diagram. These common OTUs can be seen as the core microbiota. They seem to share functional similarity in different environments and to provide a robust microbial ecosystem under environmental fluctuation (Lemanceau et al., 2017; Zorz et al., 2019). We paid close attention to the core species because they were deemed to reflect the stability of soil communities in wetlands (Yan et al., 2020). This result indicates that the methanogenic community has high potential to adjust in response to changes in vegetation type and season. Although there were only 47 core OTUs, the corresponding species accounted for a large proportion of methanogenic community composition. The similarity in the soil methanogenic populations found to exist in different wetland types and seasons signifies that these populations could be resilient and were able to quickly return to their original abundance distributions as soon as disturbances occurred in the environment. This also indicates that these methanogens in dynamic wetland environments were likely to generate the ability to adapt to such disturbances (Milferstedt et al., 2010). Although similar sequences were detected among different wetlands, each wetland has its own characteristic and distinctive OTU types, which suggests that methanogen communities were disturbed either by changes in vegetation type or by season.

Seasonal variations in climate may have considerable impact on methanogenesis in wetland ecosystems and are also an underlying driving factor affecting the structure of methanogenic communities (Kim et al., 2012; Lewa & Glińska‐Lewczuk, 2018; Xiao et al., 2017). Methanogens are sensitive to seasonal variation due to changes in oxygen availability and temperature (Seo et al., 2014), and thus, the abundance and composition of methanogens are very variable in wetland ecosystems. In the current study, the results showed that the distribution of Methanobacteriaceae (hydrogenotrophic methanogens) was higher in winter in each wetland (Fu et al., 2015; Xu et al., 2012), while Methanosarcinaceae and Methanosaetaceae accounted for a higher proportion in summer. The variation in methanogenic community structure also gave rise to a shift in potential methanogenic metabolic pattern. Previous studies have demonstrated that hydrogenotrophic methanogen can exist under psychrophilic conditions (Falz et al., 1999), which agrees with the findings of this research. Season altered methanogenic metabolic pattern. It can be clearly seen that the hydrogenotrophic pathway played a fundamental role in methanogenesis in each wetland. This also indicated that Cluster I, phylogenetically assigned to Methanobacteriaceae and its closest relatives, was the major population in all three wetland sites. Meanwhile, although most of the results of the α‐diversity indices were higher in winter than in summer, we found no statistically significant difference in the richness and diversity of the methanogenic communities among the different samples. Given the toxic effect of oxygen on methanogens, it is possible that snow and ice cover formed a diffusive barrier to the air in winter (Milferstedt et al., 2010; Vigneron et al., 2019). Wetland soils were therefore in anaerobic conditions, which was beneficial to raise the diversity of the soil methanogenic community. In this study, more unique OTUs were detected in winter, which further supports this conclusion.

Wetland vegetation species could also be an important factor driving the variation in methanogen community composition and methanogenesis (Andrews et al., 2013; Zhang, Liu, et al., 2018; Zhang et al., 2019). We found differences in the abundance distribution and composition of the methanogenic communities according to wetland type. The soil methanogen populations in each vegetation type were unique, while differences in the vegetation type resulted in the observed discrepancies in methanogenic composition. The higher relative abundance of Methanobacteriaceae and Methanosaetaceae in *A. sibirica* and *B. ovalifolia* wetlands presented an obvious difference in vegetation pattern compared with the *B. platyphylla*–*L. gmelinii* wetland, in which higher relative abundance of Methanosarcinaceae and Methanomassiliicoccaceae was found. Species of Methanosarcinaceae, many of which can possess both hydrogenotrophic and acetoclastic methanogenesis (Heli et al., 2015), had a markedly higher abundance in the *B. platyphylla*–*L. gmelinii* wetland during the summer than other wetlands. Zhang, Liu, et al. (2018) also confirmed that the number and percentage of methanogens in a constructed wetland were affected by the plant species present. Different characteristics of plants, such as the root exudates, litters, and root porosity, can induce competition in microbial communities (Sutton‐Grier & Megonigal, 2011; Zhang, Liu, et al., 2018). Furthermore, the available electron donors and acceptors in the soil are influenced by plants species, which causes changes in the structural variation of the methanogenic communities and that of other microbes (Sutton‐Grier & Megonigal, 2011).

The results of the β‐diversity analysis, such as cluster analysis and PcoA, demonstrated that vegetation type was the primary factor affecting the variation observed in the composition of the methanogenic community. LEfSe analysis also revealed differences in the methanogenic taxa from phylum to genus level according to wetland vegetation type. The result demonstrated that the number of Methanosaetaceae members in the *B. ovalifolia* wetland was significantly higher than that in the other wetlands, indicating that the aceticlastic methanogenesis pathway (which is regulated by family Methanosaetaceae) occupied a significant place in this wetland. Meanwhile, the order Methanobacteriales, which regulates the hydrogenotrophic methanogenesis pathway, had a significantly higher distribution in the *A. sibirica* wetland. In addition, there was a considerable number of unknown taxa in this wetland, which need to be further explored. The relative abundance of the Thermoplasmata group (seven taxa) in the *B. platyphylla*–*L. gmelinii* wetland was significantly higher than that in the other wetlands. Methanomassiliicoccales, as a subgroup of Thermoplasmata, is phylogenetically distant from the other methanogens (Borrel et al., 2014). Also known as Methylotrophic methanogens, Methanomassiliicoccales can metabolize methanol (Dridi et al., 2012; Yang et al., 2020) and mostly originated from animal intestinal and rumen tracts (Jin et al., 2017; Söllinger et al., 2016). As supported by the findings of this study, they are generally less distributed in environmental ecosystems due to limited methylic precursors (Borrel et al., 2011; Ren et al., 2018). Nevertheless, Methanomassiliicoccales or unclassified Thermoplasmata‐like species can utilize the noncompetitive methyl compounds as a preferred substrate to participate in the synthesis of methane (Oremland & Polcin, 1982), when acetoclastic and hydrogenotrophic methanogenesis are hindered (Rissanen et al., 2017). The contribution of Thermoplasmata with less distribution in *B*. *platyphylla*‐*L*. *gmelinii* wetland soil as a characteristic metabolic pathway can be inestimable, compared with other wetlands. These results indicate that wetland vegetation type significantly influenced the composition of soil methanogens and further created the conditions for different biochemical metabolic patterns for methanogenesis.

To investigate correlations between key methanogenic species, a network analysis was built and was used to determine whether their relationships are based on sharing or competition within complex co‐occurrence patterns. Network analysis indicated strong links between methanogens, with significant positive or negative associations (276 edges) among 51 OTUs (nodes) from eight families (*p* < .05). These OTUs were correlated with hydrogenotrophic (Methanobacteriaceae and Methanocellale), acetoclastic (Methanosaetaceae), facultative (Methanosarcinaceae), and methylotrophic (Methanomassiliicoccales and unclassified Thermoplasmata) metabolizing methanogens. In the network, hydrogenotrophic methanogenic lineages, such as members of the Methanobacteriaceae with 31 nodes (OTUs), were the most abundant, and strong positive and negative connections both within and between families were also present. OTU 1823, as the most abundant OTU in Methanosarcinaceae (facultative methanogenesis), was correlated negatively with some OTUs in Methanosaetaceae (acetoclastic methanogenesis) and a portion of OTUs in Methanobacteriaceae (hydrogenotrophic methanogenesis). Meanwhile, OTU 2174, OTU 1595, and OTU 72 in Methanosaetaceae also showed a negative correlation with some OTUs in Methanobacteriaceae. The previous study discovered that acetate could be degraded to produce CH_4_ by either aceticlastic methanogenesis or by syntrophic acetate oxidation (Kato et al., 2014). The aceticlastic pathway was only regulated by aceticlastic methanogens, whereas the syntrophic acetate oxidation pathway was mediated by the synergistic effect of two kinds of microbes (Jetten et al., 1992; Zinder & Koch, 1984). First, acetate was oxidized to H_2_ and CO_2_ by syntrophic acetate‐oxidizing bacteria and was then further metabolized to methane by hydrogenotrophic methanogens. Therefore, competition not only occurred among different trophic microorganisms for population niches, but could also exist between species on the common substrate. Nevertheless, coexistence among various microorganisms with similar trophic niches had an important influence in maintaining the functional stability and resilience of microbial ecosystems (Deng, 2012; Loreau et al., 2001). In this study, the stable coexistence of methanogenic core members in different seasons and in different vegetation types also showed that both positive and negative interspecies interactions among members support the stability of these functional communities.

The characteristics of methanogenic community structure and metabolism according to different vegetation types and seasons may be further represented by methanogenic indicator species. In the current study, methanogenic indicator OTUs can be largely divided into four types of methanogenic metabolic pattern: hydrogenotrophic, acetoclastic, facultative, and methylotrophic. Nine indicator OTUs represented the *B. platyphylla*–*L. gmelinii* wetland in summer and belonged to three methanogenic metabolic patterns. Unclassified Thermoplasmata*,* as an indicator OTU, was detected only in the *B. platyphylla*–*L. gmelinii* wetland in summer, which induced more diverse methanogenic metabolic patterns. *A. sibirica* is a vegetation type in the transition zone from forested wetland to shrub wetland. In the current study, the soil methanogenic community composition of *A. sibirica* was similar to that of *B. ovalifolia*, which represents shrub wetland. All indicator OTUs of *A. sibirica* and *B. ovalifolia* for the summer were classified to Methanosaetaceae. The number of indicator OTUs in these two wetlands was higher in winter than in summer and mainly belonged to Methanobacteriaceae. Some researchers also consider that methanogenesis is implemented by catabolic interactions among microorganisms at different trophic levels (Kato & Watanabe, 2010; Kato et al., 2014). Thus, each vegetation type has manifold indicator OTUs which represent the characteristic methanogenic metabolic patterns in different seasons. This paper reveals a new view of the transformation of soil methanogenic metabolic patterns across the transition zone of high‐latitude forested wetland.

## 5 CONCLUSIONS

In summary, we detected that soil methanogenic communities in high‐latitude natural wetlands of China have high sensitivity to season and vegetation type. Compared with season, vegetation type displayed a stronger influence on the establishment of a methanogenic community. Changes in methanogenic communities further affected the shifts in methanogenic metabolic patterns. Our results also reflected that the coexistence of multiple metabolic patterns and a steady core community enhanced the resilience of methanogenesis processes to environmental changes. The construction of a network was also useful for assessing symbiotic or competitive interspecies interactions in wetland environments. Thus, we suggest that vegetation type should be taken into consideration when selecting appropriate sites for the restoration of forested wetland in order to optimize environmental benefits and reduce methane production.

## CONFLICT OF INTEREST

The authors declare that they have no conflict of interest.

## AUTHOR CONTRIBUTIONS

**Di Wu:** Data curation (equal); Funding acquisition (equal); Funding acquisition (equal); Visualization (equal); Visualization (equal); Writing‐original draft (lead). **Caihong Zhao:** Data curation (equal); Methodology (equal). **Hui Bai:** Data curation (equal); Funding acquisition (equal); Methodology (equal). **Fujuan Feng:** Funding acquisition (equal); Writing‐review & editing (equal). **Xin Sui:** Methodology (equal); Visualization (equal); Writing‐review & editing (equal). **Guangyu Sun:** Methodology (equal); Supervision (equal); Writing‐review & editing (equal).

## Supporting information

Supplementary Material

## Data Availability

All methanogenic raw sequences have been deposited in the NCBI Sequence Read Archive (No. SRP273641).
