# Supplementary material for: Characteristics and metabolic patterns of soil methanogenic archaea communities in the high‐latitude natural forested wetlands of China
Source: Ecol Evol. 2021 Jul 4;11(15):10396–408. doi: 10.1002/ece3.7842 (PMC8328403; doi:10.1002/ece3.7842)
Supplement: Supplementary file 1 — Supplementary Material [file ECE3-11-10396-s001.pdf]

Supporting information

Characteristics and metabolic patterns of soil methanogenic archaea communities in the high latitude natural forested wetlands of China

Di Wu[1,2], Caihong Zhao[2], Hui Bai[3], Fujuan Feng[2], Xin Sui*[4], Guangyu Sun[1,2]**

[1]Key Laboratory of Saline-alkali Vegetation Ecology Restoration (Northeast Forestry University), Ministry of Education, Harbin 150040, China
[2]College of Life Science, Northeast Forestry University, Harbin 150040, China
[3]Key Laboratory of Fast-Growing Tree Cultivating of Heilongjiang Province, Forestry Science Research Institute of Heilongjiang Province, Harbin 150040, China
[4]Heilongjiang Provincial Key Laboratory of Ecologial Restoration and Resource Utilization for Cold Region, School of Life Sciences, Heilongjiang University, Harbin 150080, China
* **Corresponding author**: E-mail: xinsui_cool@126.com
** **Corresponding author:** E-mail: sungy@vip.sina.com

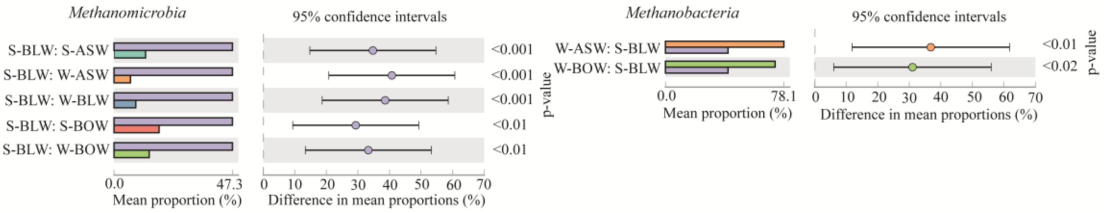

Figure S1 The significance test of the relative abundance of soil methanogenic community at class level from different wetlands.

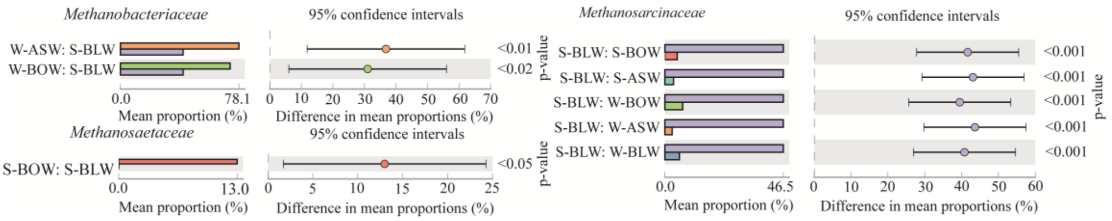

Figure S2 The significance test of the relative abundance of soil methanogenic community at family level from different wetlands.

Table S1 One-way ANOVA of the total relative abundance of the core OTUs at class level among different wetlands.

Source	SS	df	MS	F	P
Between Groups	0.400	5	0.080	20.811	<0.001
Within Groups	0.046	12	0.004		
Total	0.446	17			

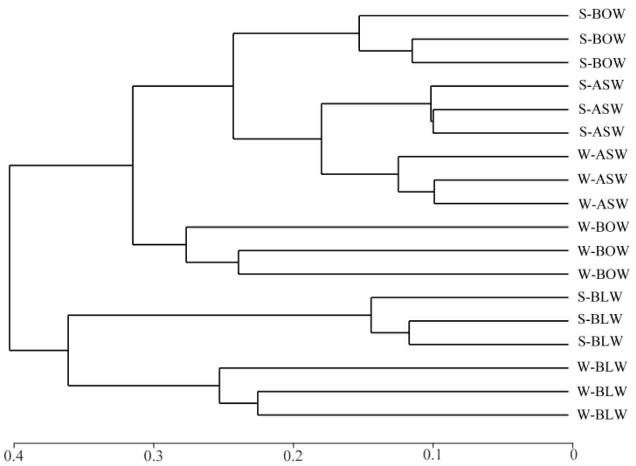

Figure S3 Cluster analysis of soil samples from different wetland soils at the OTU level.
